# The role of expression and polymorphism of the BAG-1 gene in response to platinum-based chemotherapeutics in NSCLC

**DOI:** 10.3892/or.2011.1591

**Published:** 2011-12-13

**Authors:** YA-DI WANG, MIN-WEN HA, JIAN CHENG, WEN-LU ZHANG, XUE CONG, CHUN-YAN TONG, JING SUN

**Affiliations:** 1 Liaoning Medical University, Jinzhou 121000; 2 Department of Oncology, The First Affiliated Hospital of Liaoning Medical University; 3 Dalian Medical University, Dalian 116000, Liaoning; 4 The Affiliated Hospital of Binzhou Medical College, Binzhou 256200, Shandong, P.R. China

**Keywords:** BAG-1, non-small cell lung cancer, platinum-based chemotherapeutics, sensitivity, genotype, RT-PCR

## Abstract

We investigated the correlation between BAG-1 expression and sensitivity to platinum-based chemotherapeutics in patients with non-small cell lung cancer (NSCLC). mRNA and protein expression of BAG-1 in lung tissue of NSCLC postoperative patients (I–IIIA stage) or healthy subjects were detected using reverse transcription polymerase chain reaction (RT-PCR) and immunohistochemistry, respectively. Cox regression analysis was used to quantify the association of prognostic factors with survival in NSCLC patients. Venous blood samples from patients newly diagnosed with advanced NSCLC (IIIB–IV stage) were collected before chemotherapy to analyze allelic frequency and gene polymorphisms. Compared to healthy controls (11.67%, 14 cases), levels of mRNA and protein of BAG-1 in lung tissues was significantly higher in NSCLC patients (61.67%, 74 cases) (χ^2^=5.601, P<0.05). Moreover, BAG-1 expression was identified as an independent prognostic factor for survival in NSCLC patients. As time to progression and survival rate was dramatically increased, patients with a positive expression of BAG-1 exhibited a prolonged survival period (TTP, 49.3 months; 5-year survival rat, 16.21%) compared with those without BAG-1 expression (χ^2^=7.243, P<0.05). Two BAG-1 digestion patterns (CC and CT) were identified and confirmed. Patients (77.46%) had a C/C genotype at BAG-1 codon 324, while 22.54% had the C/T genotype. The T/T genotype was not present in these patients. The progression risk of patients carrying the C/C genotype at *Bag-1* codon 324 was 1.87 times higher than that of patients carrying the C/T genotype (P<0.001). Follow-up examination showed that the chemotherapeutic sensitivity of patients carrying the C/C genotype was 2.852 times higher than that of patients carrying the C/T genotype (95% CI, 1.133–7.182; P=0.026). Significant differences were found in the median progression-free survival (PFS) and overall survival (OS) of these two cohorts of patients. Compared to patients carrying the C/T genotype of BAG-1, patients carrying the C/C genotype at *Bag-1* codon 324 exhibited better responses to platinum-based chemotherapy. Hence, the expression of BAG-1 was closely associated with the sensitivity to platinum-based chemotherapeutics in NSCLC patients.

## Introduction

Lung cancer is a leading cause of cancer-related deaths in both men and women worldwide (1). Approximately 80% of these patients represent non-small cell lung cancer (NSCLC). Without treatment, the median survival of patients with NSCLC is 4–5 months, with a survival rate at one year of 10% (2). Chemotherapy is recognized as efficient treatment for patients with advanced NSCLC since it reduces symptoms and improves the quality of life (3,4). Importantly, standard regimens of platinum-based chemotherapy resulted in a median survival of ~10 months (5). However, low response rates to platinum-based chemotherapy in patients with advanced NSCLC were observed due to the resistance to platinum compounds (6).

BAG-1 is an anti-apoptotic protein which binds to a variety of cellular proteins and modulates their intracellular functions (7–9). Moreover, overexpression of BAG-1 has been found in many forms of cancers, including breast, lung, squamous cell carcinomas and glioblastoma (10). Enhanced expression of BAG-1 was also detected in tumor samples obtained from patients with NSCLC (11). However, the involvement of BAG-1 in the tumor development and chemotherapy in NSCLC patients has not been clarified.

Particular gene polymorphisms may modify the susceptibility to NSCLC development, which includes the 308 G/A and 238 G/A polymorphisms in the promoter region of TNF-α, the cyclin D1 (CCND1) A870G gene polymorphism and the matrix metalloproteinase (MMP) 3 promoter polymorphism (12–14). DNA sequencing analysis exhibited that C/T transition occurred in exon 7 of BAG-1 gene codon 324, leading to changes in the encoded amino acid from Thr to Ile (15). Such gene polymorphisms may affect the susceptibility to tumor development. Hence, characterization of a genetic profile unveils the critical role in contributing to the definition of a better chemotherapy treatment.

In the present study, elevated BAG-1 expression was found in the tumor tissues of patients with NSCLC. This study also advanced NSCLC patients by examining the blood of Bag-1 expression in various genotypes on the efficacy of chemotherapy regimens containing different platinum. The genotype at C/C genotype at *Bag-1* condon 324 was closely correlated with the sensitivity to platinum-based chemotherapeutics in advanced NSCLC patients. These observations provide a theoretic basis for the design of individual chemotherapies for NSCLC patients.

## Materials and methods

### Patients

A total of 120 patients who underwent NSCLC surgery (I–IIIA stage) were selected from the First Affiliated Hospital of Liaoning Medical University between May 2004 and March 2006. Clinicopathological features of these patients are summarized in Table I. Samples used for immunohistochemistry and reverse transcription polymerase chain reaction (RT-PCR) were obtained from these patients. In China, NSCLC accounts for 80–85% of lung cancers and most patients (>70%) at the time of diagnosis are at unresectable IIIB or IV stage. In order to analyze the allelic frequency and gene polymorphism of *Bag-1* at codon 324, 142 patients with newly diagnosed advanced NSCLC (IIIB–IV stage) assessed via bronchofiberscope or exfoliative cytological were included (Table IV). Since small amounts of pathological tissues obtained by puncture were not enough for the follow-up study, venous blood samples were collected prior to chemotherapy. All patients with a Karnofsky performance status (KPS) score ≥70 had solid tumors, which were confirmed by computed tomography (CT) or magnetic resonance imaging (MRI). Blood, liver and renal functions and electrocardiograms were within normal range. Informed consent was obtained from all participants who met eligibility criteria. The ethics committee at the First Affiliated Hospital of Liaoning Medical University approved this study.

### Chemotherapy

Sixty-six cases were treated with an intraperitoneal (IP) injection of cisplatin (DDP) (30 mg/m^2^) for 2–4 days and vinorelbine (NVB) (25 mg/m^2^) on Days 1 and 8. Seventy-six patients were treated with IP injections of DDP (30 mg/m^2^) for 2–4 days and paclitaxel (TAX) (175 mg/m^2^) on Day 1. The procedures were repeated every 3 or 5 weeks.

### Immunohistochemistry and scoring methods

BAG-1 expression in tumor tissues from patients was detected using immunohistochemistry analysis. The 10% formalin-fixed and paraffin-embedded tissue sections were stained with anti-BAG-1 antibody at a dilution of 1:150 (Santa Cruz Biotechnology, Santa Cruz, CA). Immunohistochemical analysis was performed with a two-step immunohistochemistry detection kit (PV-6000-G) according to the manufacturer's instructions (Beijing Zhong Shan-Golden Bridge Biological Technology Co., Ltd., Beijing, China). PBS was used instead of BAG-1 primary antibody as negative control. Micrographs were analyzed by microscopy image technology. BAG-1 mainly distributed in nuclear, showing brown-yellow or dark brown vesicles. Five fields were randomly selected under microscope and the immunostaining was scored as previously described (6). The intensity of immunostaining was scored as: 0, negative staining; 1, light yellow; 2, brown-yellow and 3, dark brown. The percentage of the immuno-positive cells was assigned to one of five categories: 0, negative staining; 1, ≤10%; 2, 11–50%; 3, 51–75%; and 4, >75.0%. The weighted score for each tumor specimen was determined by multiplying the percentage score by the intensity score. The weighted score ≥3 was recognized as positive.

### RT-PCR

Total RNA was isolated using a kit (Tiangen Biotech Beijing Co., Ltd.) from 50 mg tissue sample according to the manufacturer's instructions. cDNA was then transcribed with the application of Thermo M-MLV reverse transcriptase, Oligo (dT) and dNTP (Takara). Specific primers for BAG-1 or β-actin were designed using the Primer 5.0 software and were synthesized by Takara Biotechnology (Dalian, Co., Ltd.) as follows: Bag-1, forward: 5′-GTTG TCAGCACTTGGAATAC-3′; reverse: 5′-AGATGTTCTG CTCCACTGT-3′; β-actin, forward: 5′-AAGTACTCCGT GTGGATCGG-3′; reverse: 5′-ATGCTATCACCTCCCC TGTG-3′. PCR reaction was carried out in a final reaction volume of 25 μl using the following conditions: a preheating cycle at 94˚C for 5 min, followed by 35 cycles at 94˚C for 30 sec, 53˚C for 50 sec, and 72˚C for 35 sec and finally elongated at 72˚C for 10 min. The expected products were separated on a 1% agarose gel.

### PCR-restriction fragment length polymorphism (PCR-RFLP) analysis

Prior to chemotherapy, 3–5 ml of venous blood was collected and DNA was extracted using AxyPrep Multisource Genomic DNA Minprep kit obtained from Axygen Biotechnology Ltd. (Hangzhou, Zhejiang, China). Specific primers for BAG-1 codon 324 (synthesized by Takara Biotechnology Dalian Co., Ltd.) were forward: 5′-CAGGTAGTGTAGGAGCGTGGTG-3′; and reverse: 5′-CACCCAGAGGTCCAAACAGC-3′. PCR reactions were carried out in a final reaction volume of 20 μl using the following conditions: a preheating cycle at 95˚C for 5 min, followed by 35 cycles at 95˚C for 30 sec, 63˚C for 45 sec, and 72˚C for 45 sec and finally elongated at 72˚C for 10 min. The PCR products were digested using HpyCH4III restriction enzyme (New England BioLabs) and separated on a 3% agarose gel.

### Therapeutic evaluation and survival analysis

Therapeutic effect was evaluated two or three weeks after chemotherapy according to the Response Evaluation Criteria In Solid Tumors (RECIST): complete response (CR), disappearance of all target lesions, confirmed at ≥4 week; partial response (PR), ≥30% decrease from baseline, confirmed at 4 weeks; progressive disease (PD), ≥20% increase over the smallest sum observed, or appearance of new lesions; stable disease (SD), neither PR nor PD criteria were met. CR plus PR was recognized as a response, while SD+PD had no response. The overall survival of individual patients was defined from the day of surgery up to the last follow-up examination (July 31th, 2010). Median progression-free survival (PFS) and overall survival (OS) were plotted.

### Statistical analyses

Statistical analyses were performed using SPSS 13.0 software. Data were presented as mean ± standard deviation (SD). P<0.05 was considered to be statistically significant. Comparisons between the two groups of subjects were performed using a χ^2^ test. Kaplan-Meier analysis was used to estimate survival. Differences between factors were evaluated using the log-rank test. Cox regression was used for determining prognostic factors.

## Results

### Expression of BAG-1 in lung tissues of NSCLC patients

**Figure 1:**
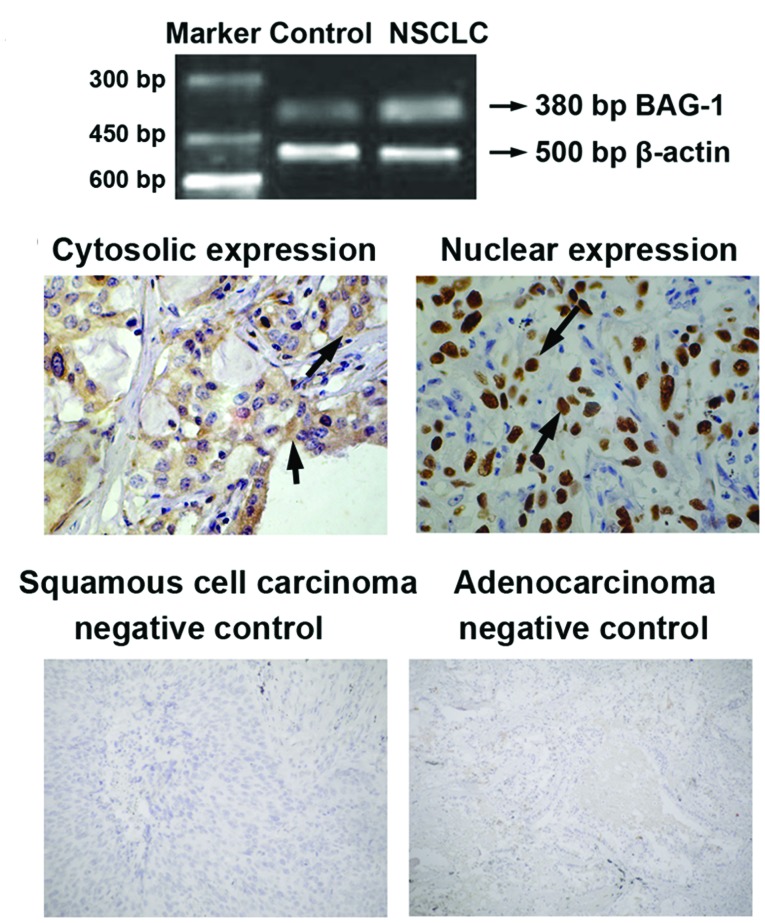
mRNA and protein expression of BAG-1 in lung tissues of NSCLC patients in I–IIIA stage. (A) Total RNA was isolated from lung tissues and RT-PCR was performed. Agarose gel eletrophoresis of PCR products were shown as indicated. Maker DL1000 was used to indicate PCR product lengths. Lung tissue samples obtained from healthy subjects were applied as control. (B) The 10% formalin-fixed and paraffin-embedded lung tissue sections were stained with anti-BAG-1 antibody. Immunoreactivity was visualized using an SP immunohistochemical staining kit according to the manufacturer's instructions. Immunohistochemical analysis showed cytosolic and nuclear expressions of BAG-1 in lung tissues of NSCLC patients. Negative controls for specimens obtained from patients with squamous cell carcinoma and adenocarcinoma are presented.

RT-PCR and immunohistochemistry were performed to examine the expression of BAG-1 in lung tissues of NSCLC patients and healthy subjects. As shown in Fig. 1A, enhanced BAG-1 mRNA level was detected in lung tissues of NSCLC patients, whereas a relatively low level of BAG-1 mRNA was found in lung tissues obtained from healthy controls. In addition, immunohistochemical analyses found that most of the BAG-1 protein was located in the cytoplasm, although nuclear staining of BAG-1 was also observed (Fig. 1B).

### Relationship between BAG-1 expression and clinicopathological characteristics

We next evaluated the potential correlations between BAG-1 expression and clinicopathological characteristics of NSCLC patients. As shown in Table I, BAG-1 expression was closely related to differentiation stage, as elevated BAG-1 expression was found in patients with moderate/high differentiation as compared with poor differentiation (P=0.031). However, no statistically significant difference in BAG-1 expression was found in the various genders, ages, pathological types, clinical stages or node metastases (P>0.05, Table I). Statistical analyses showed that 11.67% (14 cases) of healthy subjects and 61.67% (74 cases) of NSCLC patients expressed both mRNA and BAG-1 protein in the lung. BAG-1 expression was significantly higher in NSCLC patients than in healthy controls (χ^2^=5.601, P<0.05).

### Survival analysis

**Figure 2:**
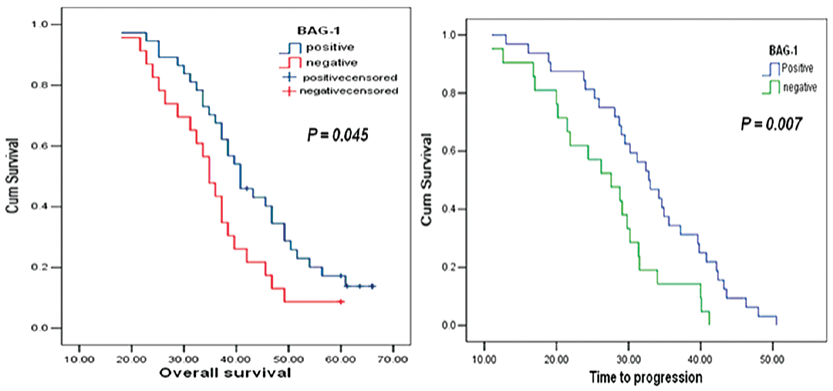
Survival curves of patients. (A) The overall survival of NSCLC patients with BAG-positive or -negative expression (χ^2^ =4.032, P=0.045). (B) The time to progression of NSCLC patients with BAG-positive or -negative expression (χ^2^=7.243, P=0.007).

Multivariate analyses (Cox regression model) were used to examine the correlation between prognostic factors and survival in NSCLC patients. As shown in Table II, differentiation stage, clinical stage as well as BAG-1 expression were identified as the independent prognostic factors for survival in patients with NSCLC (P<0.05). Overall survival (OS) was counted from the day of surgery to the last follow-up examination and was longer with BAG-1 positive as compared with BAG-1 negative (log-rank test, P=0.045) (Fig. 2A). Life-table analysis showed that the time to progression (TTP) of NSCLC patients with BAG-1 negative expression (46 cases) was 37.5 months and the 5-year survival rate was 8.69%. However, both TTP and the survival rate of NSCLC patients with BAG-1 positive expression (74 cases) were dramatically increased (TTP, 49.3 months; 5-year survival rate, 16.21%) (χ^2^=7.243, P=0.007) (Fig. 2B). These results suggested that BAG-1 expression was positively associated with prolonged survival of patients with NSCLC.

### Evaluation of genotype frequencies and Hardy-Weinberg equilibrium testing

**Figure 3:**
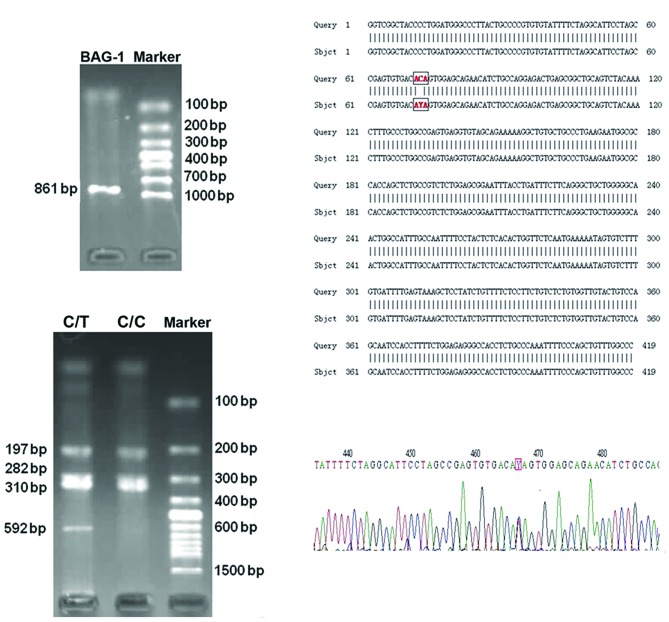
Two genotypes were identified at BAG-1 condon 324 in 142 patients with newly diagnosed advanced NSCLC (IIIB–IV stage). (A) PCR amplification product of BAG-1 DNA (861 bp). Marker, DL1000 DNA marker. (B) PCR products were digested by HpyCH4III restriction enzyme and separated on a 3% agarose gel. Marker, DNA marker 100 bp. (C) DNA sequencing of the PCR products. (D) Wave curves of DNA sequencing. Note that there was one single nucleotide mutation in the boxed site. Y stands for the C, T bases.

A total of 142 patients with newly diagnosed advanced NSCLC were included for determination of genotype frequencies. Venous blood samples were collected and BAG-1 DNA (861 bp in length) was amplified using PCR (Fig. 3A). Two BAG-1 digestion patterns were identified by digestion: 72, 197, 282 and 310 bp fragments as wild homozygous CC genotype; 72, 197, 282, 310 and 592 bp fragments as heterozygous CT genotype (Fig. 3B). The PCR products were confirmed by DNA sequencing (Fig. 3C and D). In addition, 110 out of 142 cases (77.46%) had the C/C genotype at BAG-1 nucleotide codon 324, while 32 out of 142 cases (22.54%) had the C/T genotype at codon 324. The T/T genotype was not found in these patients. Each genotype observed in the NSCLC patients were not significantly different for the values expected at BAG-1 codon 324 according to Hardy-Weinberg equilibrium (χ^2^=3.598, P=0.057). This indicates that two types of polymorphisms come from the mendelian population, which is in accordance with genetic equilibrium.

### The correlation between gene polymorphism and the sensitivity to chemotherapy

The association between gene polymorphism (C/C, C/T) and the effect of chemotherapy were tested. Therapeutic effect was evaluated two or three weeks following chemotherapy according to the RECIST as described in Patients and methods. Note that the *Bag-1* polymorphisms might influence clinical outcomes to chemotherapy as NSCLC patients carrying the C/C genotype exhibited better responses to chemotherapy (Table III). However, other factors, including gender, age, smoking, pathological types, differentiation stage, clinical stage, KPS score and chemotherapy regimens, were not significantly correlated with chemotherapy sensitivity (P>0.05, Table IV). These results demonstrated that the sensitivity to chemotherapy in NSCLC patients were associated with Bag-1 polymorphisms at codon 324.

### Correlation between gene polymorphism and survival of NSCLC patients

**Figure 4:**
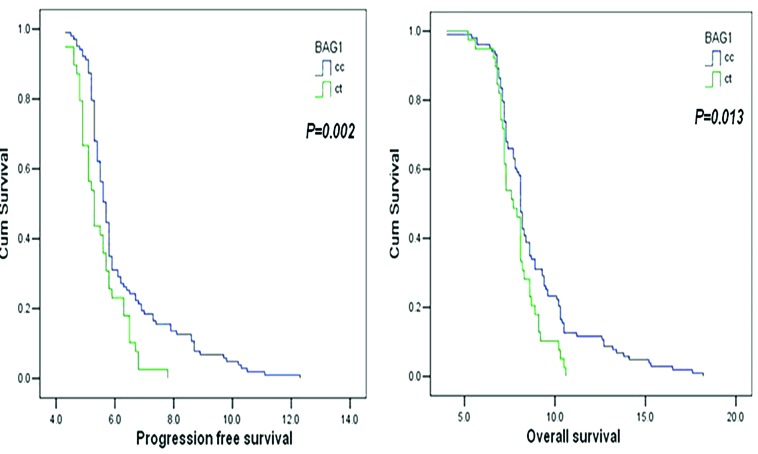
Correlation between gene polymorphism and patient survival. (A) Progression free survival of NSCLC patients carrying C/C or C/T at *Bag-1* codon 324. (B) Overall survival of NSCLC patients carrying C/C or C/T at *Bag-1* codon 324.

Kaplan Meier analyses showed that statistically significant differences were observed in both progression free survival (PFS) and overall survival (OS) of NSCLC patients carrying the C/C genotype or C/T genotype (PFS, P=0.002; OS, P=0.013) (Fig. 4). NSCLC patients carrying the C/C genotype exhibited prolonged PFS and OS as compared with those carrying the C/T genotype at *Bag-1* codon 324. Cox proportional hazards analyses further revealed that, besides gene polymorphisms, the PFS was also associated with the clinical stage and differentiation stage of patients with advanced NSCLC (Table V). Note that the progression risks of patients carrying the C/C genotype at *Bag-1* codon 324 were 1.87 times higher than in patients carrying the C/T genotype (Table V). Hence, advanced clinical stage, poor differentiation, C/T genotype at *Bag-1* codon 324 could be key factors contributing to the development of NSCLC. Moreover, we found that gender, age, KPS score were not correlated with PFS (gender, P=0.831; age, P=0.585; KPS score, P=0.155).

## Discussion

Expression of a variety of anti-apoptotic genes are involved in the development of NSCLC. Elevated Bcl-2 levels were linked to increased disease-free and overall survival (16), while decreasing Bcl-2 expression was related to metastatic NSCLC (17). Altered expression of the p53 gene was correlated with poor survival in NSCLC patients (18). The Bcl-2-binding protein BAG-1 (19), has been recognized as a multifunctional regulator of cell growth, survival and death (8,9). Overexpression of BAG-1 inhibits caspase activation induced by chemotherapeutic agents, radiation and growth factor deprivation, and therefore suppresses apoptotic cell death (20,21). However, the mechanism by which BAG-1 mediates cell survival is poorly understood. Importantly, overexpression of BAG-1 is linked to various human cancers, and may serve as an independent prognostic factor in the management of certain cancers (10). For example, BAG-1 isoforms are potential molecular markers for the pathogenesis of breast cancer (22,23). However, the correlation between BAG-1 expression and NSCLC occurrence has not been illustrated.

In the present study, we found that 11.67% (14 cases) of healthy subjects and 61.67% (74 cases) of NSCLC patients expressed both mRNA and protein of lung BAG-1. These observations were consistent with a previous report which showed overexpression of BAG-1 in 73% NSCLC patients (n=85) (11). In addition, immunostaining showed that BAG-1 protein was mostly located in the cytoplasm, although the nucleus was also observed (Fig. 1B). Studies have suggested that expression of BAG-1 in the cytoplasm might be related to a reduced risk of death in patients with NSCLC (11).

BAG-1 expression was found to be related to the differentiation of NSCLC, but not other clinicopathological characteristics, including gender, age, pathological types, clinical stage or node metastasis (Table I), suggesting BAG-1 may be involved in the progress of NSCLC and contribute to disease development. Cox multivariate analysis revealed that tumor differentiation, clinical stage as well as BAG-1 expression were independent prognostic factors for survival in patients with NSCLC (Table II). Compared with patients without BAG-1, TTP and survival rate of NSCLC patients with BAG-1 positive expression were significantly increased. This, together with another report (11), demonstrated that BAG-1 expression was sensitive to chemotherapy and could serve as an independent prognostic factor in NSCLC. However, our results demonstrating a correlation between BAG-1 expression and lung cancer patient survival was contrary to a recent study, which showed that TNM stage I lung cancer patients with BAG-1 low-expression had better survival compared to patients with high expression of BAG-1 (24). This discrepancy may be due to the different types of lung cancer studied.

Theoretically, patients with high expression of the anti-apoptosis protein BAG-1 should have poor prognosis. However, in this present study, we found that BAG-1 expression was positively associated with prolonged survival of patients with NSCLC. It is possible since the anti-apoptotic activity of tumor cells may also benefit patients with NSCLC. Tormanen *et al* analyzed 75 cases of NSCLC and revealed that enhanced apoptosis showed a 1.9-fold risk for a shortened survival in NSCLC patients (25). Nevertheless, the involved mechanism needs to be further clarified.

In addition to the anti-apoptotic effect of BAG-1 during cancer development, BAG-1 may also contribute to drug resistance of chemotherapy. In HeLa cells, down-regulation of BAG-1 conferred resistance to anti-cancer drugs, including actinomycin D, camptothecin, paclitaxel, staurosporine, thapsigargin and etoposide (26). Liu *et al* (24) reported that knock down of BAG-1 by RNA interference (RNAi) sensitized lung cancer cell lines (A549 and L9981) to cisplatin-induced apoptosis. In the present study, we found that BAG-1 expression was closely associated with the sensitivity to platinum-based chemotherapeutics in NSCLC patients, suggesting BAG-1 may be a biomarker for evaluating sensitivity to chemotherapy.

PCR-RFLP technique was applied to screen for DNA polymorphisms of BAG-1 in codon 324. C/C and C/T genotypes were detected in a total of 142 patients with newly diagnosed advanced NSCLC. NSCLC patients carrying the C/C genotype exhibited better responses to chemotherapy, with prolonged PFS and OS, as compared with those carrying the C/T genotype (Table III, Fig. 4). A previous study showed that BAG-1 is a novel regulator of nuclear factor-κB (NF-κB) as knock down of BAG-1 inhibited NF-κB activity (27). Therefore, we reasoned that the C/T transition at the BAG-1 codon 324 induced changes in encoded amino acid from Thr to Ile that contributed to alterations in protein expression and inhibition of apoptotic pathways triggered by chemotherapeutic drugs. In addition, the T/T genotype was not observed in this study, which might be due to the low incidence of two allele mutations (28). However, we cannot rule out the possibility that this may be due to the small number of patients in our study. Therefore, BAG-1 codon 324 gene polymorphism serves as a biomarker for predicting the sensitivity of chemotherapy, and may provide a theoretical basis for individual treatment of patients with advanced NSCLC.

In summary, our results indicate that BAG-1 is overexpressed in patients with NSCLC, which is associated with sensitivity to platinum-based chemotherapeutics. These results imply that BAG-1 may be a novel biomarker for predicting the sensitivity to chemotherapy and provide evidence for the application of individualized therapy in NSCLC. Future studies are needed to explore the mechanisms by which BAG-1 is involved in NSCLC progression.

## Figures and Tables

**Table I:** Relationship between BAG-1 expression and clinicopathological characteristics of NSCLC patients.

		BAG-1 expression		
			
	n	Positive	Negative	P-value
Subjects				
Healthy control	120	14	106	**<0.05**
NSCLC	120	86	34	
Benign lung tumor	10	1	9	
Gender				
Male	90	66	24	>0.05
Female	28	20	8	
Age (years)				
≤52	56	42	14	>0.05
>52	64	44	20	
Pathological types				
Squamous carcinoma	62	44	18	>0.05
Adenocarcinoma	58	42	16	
Differentiation stage				
Moderate/high differentiation	68	56	12	**<0.05**
Poor differentiation	52	30	22	
Clinical stage				
I + II	90	66	24	>0.05
IIIA	30	20	10	
Node metastasis				
With lymph node metastasis	78	50	28	>0.05
Without lymph node metastasis	42	28	14	

Data are presented as the number of cases. P<0.05 (bold text) indicates statistically significant difference.

**Table II:** Survival analysis of NSCLC patients by Cox proportional hazards model.

	F	Hazard ratio	Wald	P-value
Gender	1	1.548	1.967	0.161
Pathological types	1	0.748	0.801	0.371
Differentiation stage	1	0.505	4.518	**0.034**
Clinical stage	1	2.014	4.798	**0.028**
Node metastasis	1	1.094	0.088	0.766
BAG-1 positive	1	0.513	7.613	**0.006**

P<0.05 (bold text) indicates statistically significant difference.

**Table III:** Correlation between gene polymorphism and the effect of chemotherapy.

	The effect of chemotherapy					
					
Genotype	SD+PD cases (%)	CR+PR cases (%)	χ^2^	P-value	OR	95% CI
C/C	64	39	4.254	0.039	2.852	1.133–7.182
C/T	32	7				

SD, stable disease; PD, progressive disease; CR, complete response; PR, partial response.

**Table IV:** Association between clinicopathological factors and chemotherapeutic efficacy in NSCLC patients.

	The effect of chemotherapy			
			
Variables	SD+PD cases	CR+PR cases	χ^2^	P-value
Gender				
Male	61	28	0.095	0.758
Female	35	18		
Age (years)				
≥52	56	22	0.375	0.540
<52	40	24		
Smoking				
Yes	59	20	3.377	0.066
No	37	26		
Pathological type				
Squamous carcinoma	52	30		
Adenocarcinoma	44	16	1.136	2.286
Grade				
High	22	16	3.527	0.06
Intermidiate	38	20		
Low	36	10		
High and intermediate	60	36		
Clinical stage				
IIIB	61	35	1.699	0.192
IV	35	11		
PS				
70	30	12	1.129	0.569
80	35	15		
90	31	18		
Chemotherapy regimens				
DDP+NVB	49	17		
DDP+TAX	47	29	1.946	0.163

SD, stable disease; PD, progressive disease; CR, complete response; PR, partial response.

**Table V:** Correlation between progression-free survival and clinical stage, differentiation and gene polymorphism.

	Regression coefficient b	Standard error b	Wald	V	P-value	OR	95% CI
Clinical stage^a^	0.658	0.194	11.472	1	0.001	1.930	1.319–2.825
Differentiation stage^b^	0.353	0.117	9.120	1	0.003	1.423	1.132–1.789
C324T^c^	0.629	0.199	10.045	1	0.002	1.870	1.272–2.770

a III stage v.s. IV stage;

b moderate/high differentiation v.s. poor differentiation;

c C/C v.s. C/T.

P<0.05 indicates statistically significant difference.
